# Simulation work in adaptive dose-finding designs to identify dose-intervals that achieve targets in multiple endpoints

**DOI:** 10.1186/1745-6215-16-S2-P217

**Published:** 2015-11-16

**Authors:** James Howlett, Simon Bond, Adrian Mander, John Todd, Linda Wicker, Frank Waldron-Lynch

**Affiliations:** 1 MRC Biostatistics Unit Hub for Trials Methodology Research, University Forvie Site, Robinson Way, Cambridge, UK; 2 Cambridge Clinical Trials Unit, Cambridge University Hospitals NHS Foundation Trust Hills Road, Cambridge, UK; 3 JDRF/Wellcome Trust Diabetes and Inflammation Laboratory, Department of Medical Genetics, NIHR Cambridge Biomedical Research Centre, Cambridge Institute for Medical Research, University of Cambridge S, Cambridge, UK

## 

The DIL-frequency study aims to establish the optimal dose and repeat dosing schedule (interval) to administer ultra-low dose Interlekin-2 to participants with type 1 diabetes in order to target a desired regulatory T cell (Treg) increase and increased CD25 expression on Tregs and a minimal increase of CD4 T effector cells (Teffs).

Before the start of the trial, the available doses are chosen within a safe range. The interval is chosen to be within the range 2 to 14 days between doses. The study design has an initial learning phase with pairs of patients assigned to six pre-assigned dose-intervals. There are three further phases of eight patients, participants are given dose-interval combinations that give an expected Treg and CD25 increase closest to the targets of 20% and 25% respectively, provided that there is not an increase in Teffs above a specified level. The primary endpoints are assessed when steady state is achieved.

A variety of strategies were considered to select the dose-interval combinations for the next cohort of patients and to identify the recommended dose-interval. The method chosen was to select the dose-interval that would be closest to the target increases of Treg and CD25. The estimated coefficients from the multivariate regression analysis gave equations to predict the estimated increase in Treg and CD25 for any given dose and interval. The targets are substituted into the equations and solved to find the estimated best dose-interval. The results of the simulations are presented.

